# Parenting Style and Cyber-Aggression in Chinese Youth: The Role of Moral Disengagement and Moral Identity

**DOI:** 10.3389/fpsyg.2021.621878

**Published:** 2021-02-19

**Authors:** Yizhi Zhang, Cheng Chen, Zhaojun Teng, Cheng Guo

**Affiliations:** The Lab of Mental Health and Social Adaptation, Faculty of Psychology, Southwest University, Chongqing, China

**Keywords:** college students, parenting style, cyber-aggression, moral disengagement, moral identity

## Abstract

Previous research has shown that parenting style is intricately linked to cyber-aggression. However, the underlying mechanisms of this relationship remain unclear, especially among young adults. Guided by the social cognitive theory and the ecological system theory, this study aimed to examine the effect of parenting style on cyber-aggression, the potential mediating role of moral disengagement, and the moderating role of moral identity in this relationship. Participants comprised 1,796 Chinese college students who anonymously completed questionnaires on parenting style, moral disengagement, moral identity, cyber-aggression, and demographic variables. After controlling for sex and age, parental rejection and over-protection were positively related to cyber-aggression; however, parental emotional warmth was non-significantly related to cyber-aggression. Mediation analysis revealed that parenting style was related to cyber-aggressive behavior through moral disengagement. Moderated mediation analysis further indicated that the indirect effect of parenting style on cyber-aggression was much stronger in college students with higher moral identity. The study carries important practical implications for parents and educators concerned about the destructive consequences of cyber-aggression.

## Introduction

With the development of technology, cyber-aggression has dramatically increased in society. Cyber-aggression refers to any behavior aimed at intentionally harming individuals or groups of individuals that a person wishes to avoid (Zhao and Gao, 2012). It is enacted through digital devices such as smartphones, computers, and tablets. Cyberbullying is a subtype of cyber-aggression that has received considerable attention recently. It is focused on producing a power imbalance between perpetrators and victims (Olweus, 2013). However, research on cyber-aggression remains incipient. A previous study confirmed that despite the growing body of research regarding cyber-aggression among adolescents, studies involving young adults remain scarce (Zheng et al., 2016; Jin, 2018). In China, adolescents are forbidden to use mobile phones during school hours. However, young adults have more time and autonomy to surf the web, with college/university students comprising most of this population. Moreover, roughly 90% of all young adults devote considerable amounts of time to using the internet (Wright and Li, 2013), and the prevalence of cyber-aggression is as high as 59.47% among Chinese young adults (Jin, 2018). Importantly, research suggests that participating in cyber-aggression increases the incidence of mental health problems and suicide ideation, regardless of whether the individual acts as a perpetrator, victim, or bystander (e.g., Van Geel et al., 2014; Kowalski et al., 2016). Given the scarcity of cyber-aggression research and the adverse effects associated with this behavior, studies examining the potential risks and protective factors of cyber-aggression are necessary.

According to the ecological system theory, an individual's family forms the first social context during development, and research indicates that parenting style is closely associated with the emergence of cyber-aggressive behavior (Bronfenbrenner and Morris, 2006; Dehue et al., 2012; Rajendran et al., 2016). Parenting style comprises three dimensions: rejection, emotional warmth, and over-protection (Arrindell et al., 1999). Rejection is characterized by criticism and coldness, with such parents treating their children in a hostile, deprecating, and inattentive manner. Emotional warmth is characterized by parents who support their children, show them consideration and affection, and make them feel accepted and affirmed. Over-protective parenting is determined by a high degree of intrusion, involvement in all the child's activities, and the imposition of harsh restrictions. According to the social learning theory, the external environment contributes to the emergence and maintenance of cyber-aggressive behavior, and parenting style forms a basic model for individuals' behavior (Bandura, 1986). Moreover, previous research indicates that cyber-aggression is related to high rejection (Georgiou, 2008b; He et al., 2016, 2017), low emotional warmth (Dehue et al., 2012; Floros et al., 2013; Elsaesser et al., 2017; Moreno Ruiz et al., 2019), and high over-protection (Floros et al., 2013). Furthermore, three meta-analyses indicated that parenting style is correlated with cyber-aggression (Lereya et al., 2013; Kowalski et al., 2014; Chen et al., 2016).

However, while extant literature demonstrates that parenting style influences cyber-aggression, there is a paucity of studies regarding the underlying mechanisms that may mediate/moderate this relationship. Thus, we theorized that moral disengagement may serve as a mediating factor, and moral identity may act as a moderating factor in the direct/indirect relationship between parenting style and cyber-aggression, when controlling for sex and age.

Moral disengagement refers to specific cognitive tendencies that are used to justify immoral actions, avoid moral condemnation, and commit immoral behaviors (Bandura et al., 1996). Therefore, it can be regarded as a type of cognitive distortion. Within the social cognitive theory of morality, Bandura posited that external social contexts can activate a series of internal moral self-regulatory mechanisms which facilitate behavioral outcomes such as prosocial behavior (Bandura, 1986). However, moral disengagement can be used to selectively deactivate these moral self-regulatory mechanisms (Bandura, 1999). From this perspective, individuals who are exposed to morally disengaged attitudes may develop methods of condoning their immoral behaviors. People are not passive recipients of environmental information; instead, they actively create cognitive inferences based on environmental cues to display corresponding behaviors (Bandura et al., 1996). Moreover, the first developmental precursors of moral disengagement are experienced with one's parents, such as repeated exposure to rejecting caretaking where parents behave in morally disengaged ways. Therefore, positive parenting which employs clear limits and appropriate discussion regarding the predictable consequences of violent behavior reduces moral disengagement, and thus reduces cyber-aggression. In contrast, negative parenting characterized by unresponsive or ineffective disciplinary approaches to resolving conflicts or disputes between parents and children, and the justification of parents' harmful acts (e.g., to help children correct their mistakes), increases moral disengagement and thus increases cyber-aggression. Based on the previous delineations, we theorized that parenting style operates through moral disengagement to produce cyber-aggression.

Supporting this theoretical framework, several studies have demonstrated that moral disengagement can serve as a potential mediator among family factors (e.g., negative parenting and parental attachment), aggression, and bullying (Pelton et al., 2004; Hyde et al., 2010; Yang and Wang, 2011; Bao et al., 2015). Nonetheless, when comparing traditional aggression and bullying with cyber-aggression and cyberbullying, participation in the latter two has been shown to have greater negative effects (Bonanno and Hymel, 2013). However, to our knowledge, no prior research has examined whether moral disengagement mediates the association between parenting style and cyberbullying/cyber-aggression. Furthermore, no such study has been conducted among Chinese young adults. Additionally, there are clear cultural differences between Western and Eastern countries. For example, compared with people from Western countries, Chinese people tend to spend more time with and allocate higher value to their families (Yao et al., 2014). In sum, we believe that moral disengagement may mediate the relationship between parenting style and cyber-aggression among Chinese young adults. Two further lines of evidence can support this argument.

First, several studies have shown that parenting style is associated with moral disengagement. Additionally, while individuals with a positive parenting style may have lower levels of moral disengagement, those with a negative parenting style may have higher levels of moral disengagement (Pelton et al., 2004; Yang and Wang, 2011; Liu and Lu, 2013; Qi, 2019). Furthermore, one study showed that experiencing a rejecting parenting style at the age of 2 years positively predicted moral disengagement at the age of 15 years (Hyde et al., 2010). Research involving students in Italian elementary and middle schools also showed that poor parenting positively predicted moral disengagement 1 year later (Campaert et al., 2018).

Second, moral disengagement is an important predictor of cyber-aggression (Pornari and Wood, 2010; Wachs, 2012; Lazuras et al., 2013; Bussey et al., 2015; Yang et al., 2015, 2018; Orue and Calvete, 2016; Wang et al., 2016; Zheng et al., 2016). Consistent with this assumption, a longitudinal study showed that moral disengagement was a common antecedent for adolescents' aggressive and delinquent behavior 1 year later (Hyde et al., 2010). Moreover, five meta-analyses showed that moral disengagement was positively related to cyber-aggression (Gini et al., 2014; Kowalski et al., 2014; Wang et al., 2014; Chen et al., 2016; Killer et al., 2019).

Although parenting style may predict adults' cyber-aggression via moral disengagement, not all adults are equally influenced by parenting style. Therefore, only some adults exhibit cyber-aggressive behavior. This indicates that the relationship between parenting style and cyber-aggression may be moderated by individual characteristics. Accordingly, we propose that moral identity may influence this variation. Moral identity refers to the importance of morality in an individual's self-concept (Aquino and Reed, 2002). It has been firmly established by a series of studies as a positive correlate of prosocial behavior (Hardy et al., 2015; Reed et al., 2016) and as a negative correlate of antisocial behavior, including cyberbullying (Hardy et al., 2015; Kavussanu et al., 2015; Wang et al., 2017; Yang et al., 2018). Furthermore, a recent meta-analysis of almost 100 studies indicated that adolescents who highly value their moral identities are more engaged in moral behavior (Hertz and Krettenauer, 2016).

According to the social-ecological theory, the interaction between contextual and individual factors jointly predict cyber-aggression (Hong and Espelage, 2012). Additionally, the social cognitive theory posits that moral identity interacts with contextual factors to predict moral behaviors (Aquino et al., 2009). Thus, a strong moral identity enhances the accessibility of knowledge structures related to self-regulation and the promotion of moral behavior. However, in the presence of situational cues (e.g., violent video games), moral identity becomes less accessible (Kennedy et al., 2017). There is ample corroborating evidence suggesting that the interactions between moral identity and contextual factors predict aggression. For instance, the relationship between exposure to violent video games and cyberbullying varied according to moral identity levels among Chinese youth. Specifically, the association was weaker among youths with strong moral identities (Teng et al., 2020). Yang et al. (2018) empirically showed that having a higher moral identity weakened the mediating effect of moral disengagement in the relationship between interparental conflicts and cyberbullying. Some studies have also confirmed the association between moral identity and other predictors (e.g., school climate and trait anger) in cyberbullying (Hardy et al., 2015; Wang et al., 2017, 2019). However, to date, there is a lack of plausible evidence that moral identity may function as a moderating variable in the relationship between parenting style and cyber-aggression.

**Figure 1:**
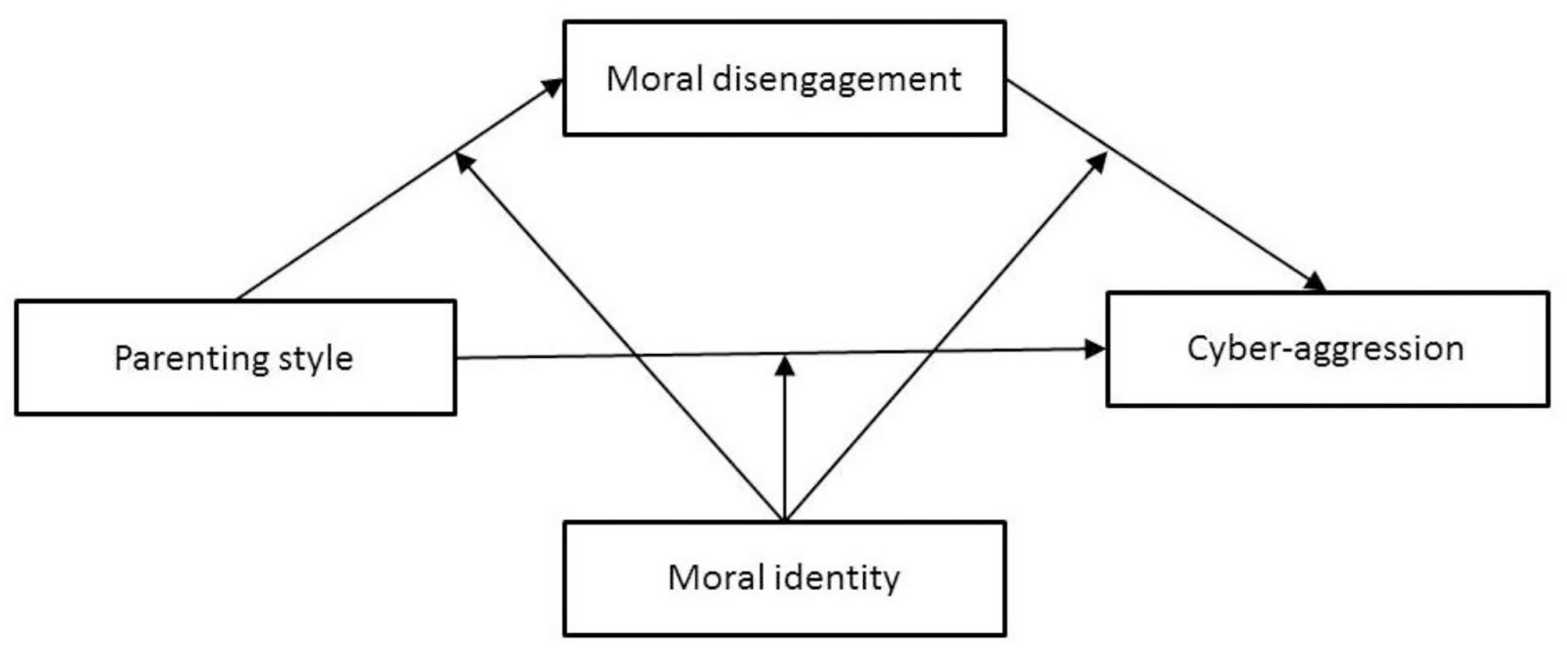
The hypothetical moderated mediation model for the associations among parenting style, cyber-aggression, moral disengagement, and moral identity.

Thus, this study aimed to further investigate the links among parenting style, moral disengagement, moral identity, and cyber-aggression. Based on the social cognitive theory, we expected moral disengagement to be a mediator in the relationship between parenting style and cyber-aggression. Furthermore, we examined whether moral identity moderated the hypothetical mediation model linking parenting style and cyber-aggression via moral disengagement (see Figure 1). Accordingly, the following hypotheses were formulated:

- **Hypothesis 1**: Parenting style relates to cyber-aggression through moral disengagement.
- **Hypothesis 2**: The direct and indirect effects of parenting style on cyber-aggression via moral disengagement are moderated by moral identity levels. Specifically, the effects will be weaker under high moral identity conditions.

## Materials and Methods

### Participants

This study was conducted at two universities in Chongqing, southwestern China. We employed a convenient cluster sampling technique to recruit 1,917 students to participate in the survey. Of these, 54 participants did not complete the surveys and were excluded from the analysis. After data cleaning, the final sample size was 1,796. The sample comprised 517 males, 1,218 females, and 61 participants of undisclosed sex. Participants' ages ranged from 16 to 27 years (*M* = 19.45, *SD* = 1.80). The sample size was calculated by G^*^Power. According to a previous meta-analysis, the relationship between positive parenting style and aggression is small yet significant (r = −0.15) (Lei et al., 2018). Accordingly, our calculations showed that a sample size of 1,796 could provide 95% statistical power to estimate the association between parenting style and cyber-aggression.

### Instruments

#### Parenting Styles

The 21-item self-reported *Egna Minnen Beträffande Uppfostran* (“My Memories of Upbringing”) short (s)-EMBU (Arrindell et al., 1999) scale was used to evaluate students' perceptions of parental rearing style. It comprised three subscales, namely rejection, emotional warmth, and over-protection. Rejection comprised seven items, with statements such as “It happened that my parents were sour or angry with me without letting me know the cause.” Emotional warmth included 6 items, such as “My parents praised me.” Finally, over protection had 10 items, including “It happened that I wished my parents would worry less about what I was doing.” The items were rated on a 4-point Likert scale ranging from 1 (never) to 4 (always). We calculated average scores for each subscale. Higher scores in each subscale indicated a more frequent use of the corresponding parenting style. The Chinese version of the scale has shown adequate validity and reliability when applied to Chinese young adults (e.g., Sun and Li, 2015). In this study, the McDonald's omega reliability coefficients for the three subscales were between 0.71 and 0.85.

#### Cyber-Aggression

The 15-item Adolescent Online Aggressive Behavior Scale (AO-ABS; Zhao and Gao, 2012) was used to assess students' online behaviors for the previous 2 months. It comprised subscales of reactive aggression and instrumental aggression. Reactive aggression included seven items (e.g., “I often abuse others when playing online games”) and instrumental aggression comprised 8 items (e.g., “I speak ill of someone with other friends on the internet”). Responses are rated on a 4-point Likert scale ranging from 1 (never) to 4 (always). Higher average scores indicate a higher frequency of cyber-aggression. For this study, the McDonald's omega coefficient was 0.92.

#### Moral Disengagement

The 32-item Moral Disengagement Scale (Bandura et al., 1996) was used to assess 8 psychological mechanisms of moral disengagement. It comprises eight subscales, each with four items. These included moral justification (e.g., “It is alright to fight to protect your friends”); euphemistic labeling (e.g., “Slapping and shoving someone is just a way of joking around”); advantageous comparison (e.g., “Stealing some money is not too serious compared to those who steal a lot of money”); displacement of personal responsibility (e.g., “A kid in a gang should not be blamed for the trouble the gang causes”); diffusion of personal responsibility (e.g., “If a group decides together to do something harmful, it is unfair to blame any kid in the group for it”); distortion of consequences (e.g., “Teasing someone does not really hurt them”); attribution of blame (e.g., “If kids fight and misbehave in school, it is their teacher's fault”); and dehumanization of victims (e.g., “Some people deserve to be treated like animals”). Responses are rated on a 5-point Likert scale ranging from 1 (strongly disagree) to 5 (strongly agree). We calculated the average score of the total items. Higher scores indicated a higher level of moral disengagement. The scale showed good validity and reliability when applied to Chinese young adults (e.g., Yang and Wang, 2012). In the current study, the McDonald's omega coefficient was 0.93.

#### Moral Identity

The 10-item Moral Identity Scale (Aquino and Reed, 2002) was used to assess the centrality of morality in students' self-concepts. It comprised two subscales, each with five items. These were internalization (e.g., “It would make me feel good to be a person who has these characteristics”) and symbolization (e.g., “I strongly desire these characteristics”). First, this scale asks participants to imagine a person who has a set of moral ideals (i.e., someone who is caring, compassionate, fair, friendly, generous, hardworking, honest, helpful, and kind). Next, participants rated the perceived level of importance of these moral ideals for each of the 10 items. Responses are rated on a 5-point Likert scale ranging from 1 (completely disagree) to 5 (completely agree). We calculated the average score of the 10 items. Higher scores indicated higher levels of moral identity. This scale demonstrated adequate validity and reliability (e.g., Aquino and Reed, 2002; Aquino et al., 2009). In the current study, the McDonald's omega coefficient was 0.81.

### Procedure

The Research Ethics Committee at Southwest University reviewed and approved this study. All participants completed the questionnaires as outlined above in classrooms after providing written and oral informed consent. Demographic information, including age and sex, was also collected. In addition, all participants were informed that the questionnaires would remain anonymous and would be used in the analysis of the present research.

### Data Analysis

We used IBM SPSS Statistics for Windows, version 23.0 to test for the missing mechanism and estimate the missing values. First, we compared the distributions of fully observed variables for a created indicator variable (1 = missing, 0 = complete) via *t*-tests and chi-square tests, with the aim of testing whether the missing data was dependent on any of the key variables (Little, 1988). Results were significant for over protection [*t*_(1838)_ = −2.50, *p* = 0.01] and age [*t*_(1839)_ = 2.09, *p* = 0.04]. This suggested that the data for these variables were missing at random (MAR), but not missing completely at random (MCAR). Findings were insignificant for rejection, *t*_(1854)_ = −1.95, *p* = 0.051; emotional warmth, *t*_(1835)_ = −1.22, *p* = 0.22; moral disengagement, *t*_(1809)_ = −0.57, *p* = 0.57; moral identity, *t*_(1838)_ = −0.34, *p* = 0.74; cyber-aggression, *t*_(1848)_ = −1.80, *p* = 0.07; and sex, χ^2^_(1,*N* = 1,800)_ = 1.37, *p* = 0.24. This suggested that these data were MCAR, but not MAR. When both MCAR and MAR occurred for the main variables, we used the maximum likelihood estimation (ML) method to fill in the missing values (Graham, 2009).

We used R software package 3.6.1 for data cleaning, since it is essential for researchers to identify invalid responses which may attenuate a study's power and increase the occurrence of type II errors in hypothesis testing (Meade and Craig, 2012). We excluded questionnaires that met any of the following conditions: the maximum number of consecutive items was equal to or greater than half the length of the total scale (Curran, 2016), the standard deviation of the last 30 items was close to zero (Meade and Craig, 2012; Dunn et al., 2018), or the correlation coefficient of psychometric synonymous items was below 0.60 (DeSimone et al., 2015).

Since the study employed the self-report method to collect data, it may have led to the common method bias effect. Therefore, an exploratory factor analysis of all items in the study was conducted to assess the severity of common method variation using the Harman single-factor test. The results showed that there were 28 factors with initial eigenvalues >1, and the largest one explained 12.79% of the total variance, which was <40%. The results of single-factor confirmatory factor analysis showed poor fit indices (χ^2^*/df* = 9.09, CFI = 0.189, TLI = 0.172, RMSEA = 0.095, SMRM = 0.116). Therefore, the common method bias in this study was negligible.

In our preliminary analyses, we used IBM SPSS Statistics for Windows, version 23.0 to calculate means, standard deviations, and Pearson correlations between interest variables, and the JASP 0.12.2 to conduct reliability analysis. In primary analyses, we used the SPSS macro PROCESS to test our models. When the skewness and kurtosis values are <2 and 7, respectively, the data distribution is accepted as normal (Curran et al., 1996). However, in our study, the distributions of cyber-aggression were skewed (skewness = 10.80, kurtosis = 235.50). The bootstrapping method does not assume normality when conducting statistical tests and constructing confidence intervals (Preacher and Hayes, 2008). Therefore, to investigate the mediation model and examine the mediating role of moral disengagement, we used PROCESS macro (model 4) with 5,000 bootstrap samples, which can provide an estimate at 95% confidence interval (CI) (Hayes, 2013). If the 95% CI of the index does not include zero, the index of the mediation is significant. We used PROCESS macro (model 59) to investigate the moderated mediation model and examine the moderating effect of moral identity on: (1) the relationship between parenting style and cyber-aggression (Model 1); (2) the relationship between parenting style and moral disengagement (Model 2); and (3) the relationship between moral disengagement and cyber-aggression (Model 3). If the 95% CI of the index did not include zero, the interaction effect was significant.

## Results

### Preliminary Analyses

Table 1 lists means, standard deviations, and the correlations among sex, age, parenting style, moral disengagement, moral identity, and cyber-aggression. As expected, cyber-aggression was positively related to rejection (*r* = 0.13, *p* < 0.01), over-protection (*r* = 0.09, *p* < 0.01), and moral disengagement (*r* = 0.22, *p* < 0.01). It was negatively correlated with emotional warmth (*r* = −0.06, *p* < 0.05) and moral identity (*r* = −0.11, *p* < 0.001). Meanwhile, both rejection and over-protection showed positive relationships with moral disengagement (*r* = 0.17, *p* < 0.01; *r* = 0.13, *p* < 0.01, respectively) and negative relationships with moral identity (*r* = −0.11, *p* < 0.01; *r* = −0.05, *p* < 0.05, respectively). Furthermore, emotional warmth showed a negative relationship with moral disengagement (*r* = −0.19, *p* < 0.01) and a positive relationship with moral identity (*r* = 0.29, *p* < 0.01). Finally, greater moral identity was associated with lower moral disengagement (*r* = −0.35, *p* < 0.01).

**Table 1:** Descriptive statistics and correlations among variables.

		**1**	**2**	**3**	**4**	**5**	**6**	**7**	**8**
1	Sex^a^	1							
2	Age	0.09^**^	1						
3	Rejection	−0.09^**^	−0.08^**^	1					
4	Emotional warmth	0.08^**^	0.03	−0.41^**^	1				
5	Over-protection	−0.07^**^	−0.13^**^	0.50^**^	−0.15^**^	1			
6	Moral disengagement	−0.22^**^	−0.09^**^	0.17^**^	−0.19^**^	0.13^**^	1		
7	Moral identity	0.11^**^	0.09^**^	−0.11^**^	0.30^**^	−0.05^*^	−0.35^**^	1	
8	Cyber-aggression	−0.11^**^	−0.06^*^	0.13^**^	−0.06^*^	0.09^**^	0.22^**^	−0.11^**^	1
*M*		0.70	19.45	1.31	3.05	2.03	1.68	4.14	1.05
*SD*		0.46	1.80	0.35	0.62	0.45	0.44	0.54	0.12

*Sex: 0 = male, 1 = female*.

* *p<0.05*,

** *p<0.01*.

### Testing for the Mediation Effect

As illustrated in Model 2 (Table 2), moral disengagement had a positive influence on rejection (*b* = 0.18, *p* < 0.001) and over-protection (*b* = 0.10, *p* < 0.001), and a negative influence on emotional warmth (*b* = −0.13, *p* < 0.001). In Model 3, both rejection (*b* = 0.03, *p* < 0.001) and over-protection (*b* = 0.01, *p* < 0.05) positively influenced cyber-aggression. However, emotional warmth did not significantly influence cyber-aggression (*b* = −0.003, *p* > 0.05). In addition, moral disengagement, which was independent of rejection, showed a positive influence on cyber-aggression (*b* = 0.05, *p* < 0.001). Moral disengagement, which was independent of emotional warmth, showed a positive influence on cyber-aggression (*b* = 0.05, *p* < 0.001). Moral disengagement, which was independent of over-protection, showed a positive influence on cyber-aggression (*b* = 0.05, *p* < 0.001). These results indicated that moral disengagement mediated: (1) the association between rejection and cyber-aggression (indirect effect = 0.009, SE = 0.002, 95%CI = [0.006,0.013]); (2) the association between emotional warmth and cyber-aggression (indirect effect = −0.007, SE = 0.001, 95%CI = [−0.009, −0.004]); and (3) the association between over-protection and cyber-aggression (indirect effect = 0.005, SE = 0.001, 95%CI = [0.003, 0.008]). Thus, Hypothesis 1 was supported.

**Table 2:** Mediation of the association between parenting style and cyber-aggression through moral disengagement.

**Predictors**	**Model 1 (Criterion: cyber-aggression)**		**Model 2 (Criterion: moral disengagement)**		**Model 3 (Criterion: cyber-aggression)**	
	***b***	***t***	***b***	***t***	***b***	***t***
Sex	−0.02	−3.91^***^	−0.20	−8.83^***^	−0.02	−2.32^*^
Age	−0.003	−1.93	−0.02	−2.92^**^	−0.002	−1.43
Rejection	0.04	4.92^***^	0.18	6.17^***^	0.03	3.84^***^
Moral disengagement					0.05	7.51^***^
**R*^2^*	0.03		0.08		0.06	
*F*	16.70^***^		47.74^***^		27.03^***^	
Sex	−0.03	−4.13^***^	−0.20	−8.75^***^	−0.02	−2.47^*^
Age	−0.004	−2.22^*^	−0.02	−3.11^**^	−0.002	−1.66
Emotional warmth	−0.01	−1.93	−0.13	−7.47^***^	−0.003	−0.53
Moral disengagement					0.05	7.91^***^
**R*^2^*	0.02		0.09		0.05
*F*	9.76^***^		53.97^***^		23.22^***^	
Sex	−0.03	−4.13^***^	−0.20	−9.05^***^	−0.02	−2.43^*^
Age	−0.003	−1.92	−0.02	−2.30^**^	−0.002	−1.42
Over-protection	0.02	2.84^**^	0.10	4.33^***^	0.01	2.05^*^
Moral disengagement					0.05	7.88^***^
*R^2^*	0.02		0.07		0.05	
*F*	11.23^***^		40.90^***^		24.26^***^	

*Each column is a regression model that predicts the criterion at the top of the column*.

* *p < 0.05*,

** *p < 0.01*,

*** *p < 0.001*.

### Testing for the Moderated Mediation

**Figure 2:**
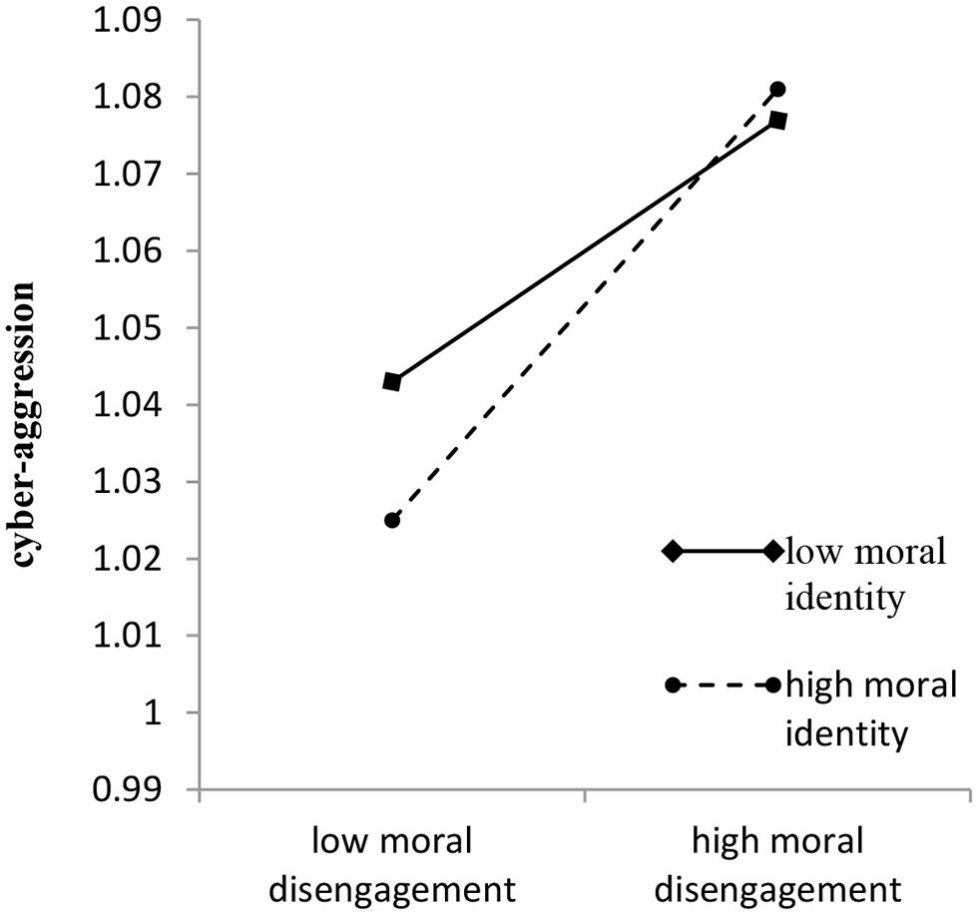
Effects of moral disengagement and moral identity on the prediction of cyber-aggression.

As illustrated in Table 3, Model 1 demonstrated that both rejection (*b* = 0.04, *p* < 0.001) and over-protection (*b* = 0.02, *p* < 0.01) were significantly related to cyber-aggression. However, emotional warmth was non-significantly related to cyber-aggression (*b* = −0.004, *p* > 0.05). Furthermore, the interactions between parenting styles and moral identity were all non-significant when predicting cyber-aggression. Model 2 indicated that rejection (*b* = 0.14, *p* < 0.001), emotional warmth (*b* = −0.06, *p* < 0.001), and over-protection (*b* = 0.09, *p* < 0.001) were significantly related to moral disengagement. However, the relationships between parenting styles and moral disengagement were not moderated by moral identity. Finally, when controlling for rejection, emotional warmth, and over-protection, the interaction between moral disengagement and moral identity in predicting cyber-aggression was significant (*b* = 0.03, *p* < 0.05; *b* = 0.02, *p* < 0.05; and *b* = 0.03, *p* < 0.05, respectively). Results of the simple slope tests showed that when moral identity was high (i.e., 1 *SD* above the mean), the positive relationship between moral disengagement and cyber-aggression was also high (*b*_simple_ = 0.64, *p* < 0.001). When moral identity was low (i.e., 1 *SD* below the mean), the positive relationship between moral disengagement and cyber-aggression was significant (*b*_simple_ = 0.37, *p* < 0.001; see Figure 2), albeit much weaker than when moral identity was high.

**Table 3:** Results of the moderated mediation model for the effects of parenting styles on cyber-aggression.

**Predictors**	**Model 1 (Criterion: cyber-aggression)**		**Model 2 (Criterion: moral disengagement)**		**Model 3 (Criterion: cyber-aggression)**	
	***b***	***t***	***b***	***t***	***b***	***t***
Sex	−0.02	−3.63^***^	−0.17	−8.02^***^	−0.02	−2.33^*^
Age	−0.002	−1.46	−0.01	−1.47	−0.002	−1.20
Rejection	0.04	4.60^***^	0.14	5.17^***^	0.03	3.76^***^
Moral identity	−0.02	−3.40^**^	−0.26	−14.08^***^	−0.01	−1.03
Rejection × Moral identity	0.01	0.93	0.05	1.07	0.003	0.24
Moral disengagement					0.05	6.94^***^
Moral disengagement × Moral identity					0.03	2.21^*^
*R^2^*	0.04		0.17		0.06	
*F*	12.30^***^		71.23^***^		16.17^***^	
Sex	−0.02	−3.85^***^	−0.17	−8.11^***^	−0.02	−2.48^*^
Age	−0.003	−1.84	−0.01	−1.84	−0.002	−1.47
Emotional warmth	−0.004	−0.85	−0.06	−3.84^***^	−0.001	−0.20
Moral identity	−0.02	−3.44^**^	−0.25	−12.88^***^	−0.01	−1.14
Emotional warmth × Moral identity	−0.01	−1.21	−0.003	−0.09	−0.01	−0.65
Moral disengagement					0.05	7.37^***^
Moral disengagement × Moral identity					0.02	2.08^*^
*R^2^*	0.02		0.17		0.06	
*F*	8.34^***^		68.25^***^		14.11^***^	
Sex	−0.02	−3.78^***^	−0.18	−8.13^***^	−0.02	−2.43^*^
Age	−0.002	−1.42	−0.01	−1.29	−0.002	−1.18
Over-protection	0.02	2.66^**^	0.09	4.02^***^	0.01	1.99^*^
Moral identity	−0.02	−3.71^***^	−0.27	−14.41^***^	−0.01	−1.19
Over-protection × Moral identity	0.01	0.71	0.03	0.76	0.001	0.12
Moral disengagement					0.05	7.21^***^
Moral disengagement × Moral identity					0.03	2.24^*^
*R^2^*	0.03		0.17		0.06	
*F*	9.44^***^		68.74^***^		14.64^***^	

*Each column is a regression model that predicts the criterion at the top of the column*.

* *p < 0.05*,

** *p < 0.01*,

*** *p < 0.001*.

Furthermore, we examined the extent to which moral identity conditionally moderated the mediation effect of moral disengagement based on the bootstrapping results. The indirect effect of rejection was significant and stronger when moral identity was high (indirect effect = 0.011, SE = 0.003, 95%CI = [0.005, 0.018]) than when it was low (indirect effect = 0.004, SE = 0.002, 95%CI = [0.001, 0.008]). Moreover, although the indirect effect of emotional warmth was significant when moral identity was high (indirect effect = −0.004, SE = 0.002, 95%CI = [−0.008, −0.001]), it was weaker and non-significant when moral identity was low (indirect effect = −0.002, SE = 0.001, 95%CI = [−0.005, 0.000]). The indirect effect of over-protection was much weaker when moral identity was low (indirect effect = 0.003 SE = 0.001, 95%CI = [0.001, 0.0060]) than when it was high (indirect effect = 0.007, SE = 0.002, 95%CI = [0.003,0.011]). Therefore, Hypothesis 2 was partially supported.

## Discussion

While previous research has demonstrated the relationship between parenting style and cyber-aggression, the underlying mechanisms of this association remain unclear. Therefore, this study aimed to examine the mediating effect of moral disengagement and the moderating effect of moral identity in the relationship between parenting style and cyber-aggression among Chinese young adults. Through the moderated mediation model proposed by the social cognitive and social ecological theories. This study suggested that parenting styles contributed to moral disengagement, which in turn, on cyber-aggression. Moreover, the mediating effect of moral disengagement in the relationship between parenting styles and cyber-aggression increased when participants have a high levels of moral identity.

### The Mediating Effect of Moral Disengagement

Consistent with previous research (Dehue et al., 2012; Lereya et al., 2013; Rajendran et al., 2016; Elsaesser et al., 2017; Moreno Ruiz et al., 2019), we found that cyber-aggression was positively correlated with rejection and over-protection. Bronfenbrenner and Morris (2006) ecological theory proposed that the family ecosystem has a significant effect on the physical, psychological, and social development of family members. Bandura (1986) social learning theory indicated that the daily interactions with parents provided a model for individuals to interact with others. That is, if parents use aggressive behaviors (e.g., quarrels, insults, and violence) to address problems and conflicts, their children may exhibit more aggressive behaviors toward others. Moreover, parents with rejecting or over-protective parenting styles are likely to neglect their children's daily behaviors and fail to effectively supervise and guide their online activities. Therefore, their children are more likely to participate in cyber-aggression (Flouri and Buchanan, 2003; Georgiou, 2008a). However, emotional warmth was not related to cyber-aggression. While numerous studies have shown that positive parenting is beneficial to children's mental health (Ok et al., 2010; Kowalski et al., 2014; Vasquez et al., 2016), a study among 180 Israeli students showed that positive parenting (i.e., autonomy-supportive parenting style) was not a positive predictor of preventing cyberbullying (Katz et al., 2019). This may be because positive parenting is not solely sufficient to prevent cyber-aggression; other factors, such as the proactive development of conflict-resolution skills and strategies, are required (Livingstone et al., 2011). However, parents who engage in the emotional warmth parenting style can guide their children's behaviors and openly discuss the risks of internet use. Therefore, their children are less likely to engage in cyber-aggression. The above-mentioned literature corroborated our results.

As expected, this study demonstrated the mediating role of moral disengagement in the relationship between parenting style and cyber-aggression. Previous studies have indicated that moral disengagement acted as a mediator in the relationship between contextual factors (e.g., school environment, violent video games) and aggression (Teng et al., 2019), including cyberbullying (Wang et al., 2019). However, to our knowledge, the current study is the first to provide evidence that the impact of parenting style on cyber-aggression is mediated by moral disengagement among Chinese young adults. In previous aggression research, the social ecological theory emphasized that ecological systems, such as one's family, served as both protective or risk factors for adolescents (Moreno Ruiz et al., 2019). However, the social cognitive theory emphasized the detrimental effect of moral disengagement (Killer et al., 2019). By integrating these two theories, we were able to explore the influence of not only parenting style on moral disengagement but also moral disengagement as a mediating variable in the relationship between parenting style (i.e., contextual factors) and cyber-aggression.

Furthermore, our findings demonstrated that parenting style was significantly related with moral disengagement, which is consistent with previous research (e.g., Pelton et al., 2004; Hyde et al., 2010). Individuals who were raised with higher levels of rejection or over-protection and lower levels of emotional warmth tend to have difficulties in feeling remorse over misconduct, which reflects the way they were treated by their parents. Accordingly, these individuals tend to gradually disengage from moral standards.

Consistent with social cognitive theory and previous studies (e.g., Bussey et al., 2015; Orue and Calvete, 2016), our research showed that moral disengagement was a useful predictor of cyber-aggression. That is, when individuals are more successful in disengaging from moral standards, they tend to feel less guilt and engage more in cyber-aggression. In sum, our study findings supported moral disengagement as a mediating mechanism through which parenting style influences cyber-aggression in adulthood.

### The Moderating Effect of Moral Identity

The present study showed that the predicted path linking moral disengagement and cyber-aggression was moderated by moral identity. Specifically, young adults with lower moral identities showed a weaker link between moral disengagement and cyber-aggression; this finding contradicts that of previous studies (Wang et al., 2017). However, our finding is congruent with the self-consistency of Blasi (1983)'s Self Model, which proposes that individuals wish to behave consistently with their self-concept. That is, if morality is central to a person's self-concept, this can evoke moral behaviors. For example, an adult with a high moral identity may deem it important to be a moral person. If this individual was to behave immorally, they would have to make greater efforts to morally disengage, compared to someone with a low moral identity. Aquino and Reed (2002) social cognitive theory of moral identity posits that a higher moral identity may cause us to focus more on the interests and needs of others (Winterich et al., 2009). Based on our findings and prior research, high moral identity is necessary to reduce cyber-aggressive behavior. Furthermore, solely reducing moral disengagement may have a relatively small impact on cyber-aggression. Thus, both enhancing moral identity and reducing moral disengagement may help to more effectively decrease cyber-aggression.

Contrary to our expectations, the first part of the mediation model (i.e., parenting style → moral disengagement) and the direct association between parenting style and cyber-aggression were not moderated by moral identity. These results contradict previous findings, which demonstrated that moral identity moderated the association among a contextual factor, moral disengagement, and aggression (Wang et al., 2019; Teng et al., 2020). This may be attributed to the presence of situational cues that can mitigate the relationship between moral identity and immoral action. For example, a study showed that the recency and a continuous reinforcement of situational activation (e.g., an immoral action) may temporarily reduce the accessibility of moral identity (Aquino et al., 2009). That is, regardless of moral identity levels, contextual factors (e.g., parenting styles) may powerfully influence moral cognition and behavior. Further research is necessary to elucidate the moderating effect of moral identity in the relationship between cyber-aggression and parenting styles.

## Limitations and Practical Implications

Our study has several limitations. First, we relied on self-reported instruments to collect data, which facilitates social desirability and common method biases. Thus, observational and experimental studies using multi-methods and evaluators (e.g., interviews) are necessary to address this shortcoming. Second, this study had a cross-sectional design, thus hindering the assessment of causality in the studied relationships. Accordingly, future longitudinal and experimental studies are necessary to confirm the reliability and effectiveness of our findings.

Nonetheless, this research also presents important practical implications for preventing and intervening in cyber-aggression. The moderated mediation developed and confirmed by us both explains how parenting style affects cyber-aggression and demonstrates when this link is most potent. Thus, interventions for the reduction of cyber-aggression in China should focus on parental education programs to raise awareness of how parents' rearing styles can influence their children's engagement in cyber-aggression. Moreover, school-level interventions must focus on strategies to decrease moral disengagement and to improve moral identity to help reduce cyber-aggression more effectively in adulthood.

## Data Availability Statement

The original contributions presented in the study are included in the article/Supplementary Material, further inquiries can be directed to the corresponding author.

## Ethics Statement

The studies involving human participants were reviewed and approved by The Research Ethics Committee at Southwest University. The patients/participants provided their written informed consent to participate in this study.

## Author Contributions

All authors participated and contributed in study design. CC collected the experimental data. YZ and ZT analyzed and interpreted the data. YZ, ZT, and CG drafted the work. Besides, all authors read and approved the final manuscript.

## Conflict of Interest

The authors declare that the research was conducted in the absence of any commercial or financial relationships that could be construed as a potential conflict of interest.
